# The Impact of Loneliness and Social Anxiety on Casual Social Contacts

**DOI:** 10.7759/cureus.70633

**Published:** 2024-10-01

**Authors:** Edward J Federman, Charles E Drebing, James E Graham

**Affiliations:** 1 Psychology, Edith Nourse Rogers Memorial (ENRM) Veterans Affairs (VA) Medical Center, Bedford, USA; 2 Psychiatry, Boston University School of Medicine, Boston, USA; 3 Psychology, VA Cheyenne Health Care, Cheyenne, USA; 4 Occupational Therapy, Colorado State University, Fort Collins, USA

**Keywords:** casual contacts, loneliness, social anxiety, social isolation, weak ties

## Abstract

Introduction

In the wake of the COVID-19 pandemic, loneliness and social isolation have become major public health crises. Loneliness has reached epidemic levels and negatively impacts both health and quality of life. “Casual contacts” is a developing line of research that may hold promise in stemming the current crisis. Casual contacts refer to interactions and relationships with people who are neither family nor friends. Our objectives are to (a) document the impact of casual contacts on community-based adults (Studies 1 and 2) and (b) study the impact of loneliness (Studies 1 and 2) and social anxiety (Study 2) on responses to casual contact. Based on prior studies, we expect to find that casual contacts have a generally positive impact on mood. However, the association of both loneliness and social anxiety with hypervigilance to social threat leads to the broad hypothesis that individuals with each of these conditions would be more likely to respond negatively to casual contacts than would others without those conditions.

Method

This correlational research recruited convenience samples of English-speaking adults living in the United States, using the online platform Survey Monkey. There were no selection criteria beyond (a) language and location and (b) the sample being generally balanced for age and gender. Partial correlation and analysis of covariance were used to examine the association of loneliness and social anxiety with feeling worse after casual contacts, while controlling for age, gender, household size, household income, and size of town. Because the current study examines a novel area, individual differences that affect how people experience casual contacts, we ran two studies enabling us to examine whether the results replicated. In total, we surveyed 546 community-dwelling adults about their casual contacts, 174 in Study 1 and 372 in Study 2. Data cleaning was used to minimize/eliminate meaningless and random answers resulting in a final total study sample of 393 participants: 123 in Study 1 and 270 in Study 2.

Results

The results indicate that casual contacts are common, with fewer than 4% having neither verbal nor electronic contact with an acquaintance weekly, and typically have a positive impact (Study 1 and Study 2). Nevertheless, a significant minority of casual contacts result in negative experiences, which is more probable among respondents who were lonely (Study 1 and Study 2) and/or had higher levels of social anxiety (Study 2). Exploratory analyses showed that both loneliness and social anxiety are linked to feeling worse for longer periods of time after casual contacts and that positive feelings tend to dissipate more rapidly for those with higher loneliness scores (Study 2).

Conclusions

These results indicate that clinicians developing interventions that involve casual contacts must consider how to do so safely and effectively for those with a higher degree of loneliness and/or social anxiety.

## Introduction

Loneliness and social isolation have become predominant public health challenges. Loneliness increases both morbidity [1] and mortality [2] while leading to declines in cognitive functioning [3]. It is associated with increased suicidal ideation and parasuicide [4] as well as completed suicides [5]. The US Surgeon General recently stated, “Given the profound consequences of loneliness and isolation, we have … an obligation, to make the same investments in addressing social connection that we have made in addressing tobacco use, obesity, and the addiction crisis” [6].

Loneliness is prevalent both in the general population and primary care patients [7]. Community surveys in the US suggest prevalence at least in the 35% to 45% range [8] and perhaps higher [6] especially since the start of the COVID-19 pandemic [9]. Prevalence rises to more alarming rates among persons with psychiatric and/or substance abuse disorders [10]. Similarly, loneliness has skyrocketed within the Veteran population, both in the US [11] and abroad [12].

Given the prevalence of loneliness as well as its powerful link to adverse outcomes, the need for effective interventions is vital. Unfortunately, the evidence supporting interventions to reduce loneliness or increase social connections is at best mixed [13,14]. Nevertheless, there is preliminary evidence to support cognitive modification for loneliness and mixed strategies that include socialization [14].

Another emerging line of research that holds promise for intervention is the potential role of casual contacts (often referred to as “weak ties”), which refers to interactions and relationships with others who are neither family nor friends (e.g., acquaintances, neighbors, coworkers). Brief interactions with casual contacts have been found to boost mood and well-being among Starbucks customers [15], in the workplace [16] and the schoolyard [17]. People typically underestimate the positive impact of casual contacts and overestimate the probability of bad outcomes in these interactions and are thus led to limit these social connections [18]. Kim [19] noted that most research examining the relationship between social connections and health comes from Western cultures and does not disaggregate strong vs. weak ties. His work in Korea found “regular interaction with weaker ties are associated with better mental health. The number of strong ties (family members and friends), on the other hand, is not a significant predictor of psychological distress” [19].

While this emerging line of research holds promise for intervention development, safe and effective interventions must reflect an understanding of how individual differences affect the experience of casual contacts. Lonely individuals have a higher social threat sensitivity than their nonlonely peers [20,21], a phenomenon already present in childhood [22]. Moreover, neuroimaging shows that lonely individuals respond more quickly to social threat stimuli [23], while an eye tracking study shows hypervigilance to social threat among lonely persons [24]. Further, the fear of scrutiny in social situations is a cardinal feature of those with social anxiety disorder [25].

Our objectives are to (a) document the impact of casual contacts on community-based adults (Studies 1 and 2) and (b) study the impact of loneliness (Studies 1 and 2) and social anxiety (Study 2) on responses to casual contact. Based on prior studies, we expect to find that casual contacts have a generally positive impact on mood. However, the association of both loneliness and social anxiety with hypervigilance to social threat leads to the broad hypothesis that individuals with each of these conditions would be more likely to respond negatively to casual contacts than would others without those conditions. Specific hypotheses are included in the Materials and Methods sections for both Study 1 and Study 2.

## Materials and methods

We investigated specific hypotheses in surveys of community samples in two studies, attempting to achieve a nationally representative sample for age, gender, ethnicity, and race. Table 1 shows these demographics for the two studies; notably 18-29 year olds are underrepresented, while 45-60 year olds are overrepresented as are females. Whites are overrepresented. When looking at only those who identify with a single race, all minority groups are underrepresented. However, if you include those who identify as more than one race, the total population of those who identify as American Indian or Alaskan Native (AIAN) is fairly represented. 

**Table 1:** Gender, age, race, and household size: Study 1 and Study 2

Measure	Study 1		Study 2	
	n	%	n	%
Gender				
Female	74	60.2	178	66.2
Male	49	39.8	91	33.8
Missing	0		1	
Age				
18-29	24	19.5	41	15.2
30-44	43	35	51	19
45-60	32	26	98	36.4
>60	24	19.5	79	29.4
Missing	0		1	
Race				
White	86	69.9	184	68.1
Black or African American	12	9.8	14	5.2
American Indian or Alaskan Native	0	0	2	0.7
Asian	6	4.9	19	7.0
Native Hawaiian or Other Pacific Islander	0	0	3	1.1
Hispanic	7	5.7	22	8.1
More than one race	12	9.8	26	9.6
Missing	0		0	
Household Size				
Live alone	15	12.2	21	7.8
1 other	31	25.2	73	27
2 others	20	16.3	68	25.2
3 others	26	21.1	52	19.3
4 others	20	16.3	24	8.9
5 or more others	11	8.9	32	11.9
Missing	0		0	

All surveys were conducted via the online service Survey Monkey, which maintains a panel of volunteers who either receive compensation or direct funds to a charity of their choice when they participate in a survey. Links to the online surveys were sent to respondents via email. To maximize data quality, Survey Monkey limits the number of surveys each individual can take and also requires panelists to regularly update their profiles. All the items in the surveys are shown in the Appendix.

Study 1

Hypotheses

1. Interaction with casual contacts will be common across a sample of adults (at least 50% will have one or more weekly) and will be frequently associated with reports of a positive impact (at least 50% will report casual contacts are positive a moderate amount of the time).

2. Participants with high scores on a loneliness scale will be more likely to report a negative reaction to casual contacts than their nonlonely peers.

Method Study 1

Participants and procedures: We recruited a convenience sample of English-speaking adults living in the US, via a panel from Survey Monkey in October 2022. There were no selection criteria beyond (a) language and location and (b) the sample being generally balanced for age and gender. Participants either received direct compensation or directed funds to a charity of their choice. Respondents were asked to complete a survey containing three demographic questions, seven survey questions, and one open-ended question related to social setting and casual contacts. Gender and age information were supplied by Survey Monkey.

Data analysis: Hypothesis 1. Descriptive statistics were used to show both the frequency of casual contacts and how often and to what extent they were associated with positive outcomes. Hypothesis 2. The association of loneliness with feeling worse after casual contacts was assessed by (a) partial correlation and (b) an analysis of covariance with loneliness as the independent variable and the frequency of feeling worse after casual contacts as the dependent variable. In each case, five variables were controlled for age, gender, household size, household income, and size of town.

There were 174 respondents. Initial review of the open-ended responses showed that some respondents had responded to this question with nonsense responses, perhaps to ensure they received compensation. To guard against using data from inattentive or careless subjects, we reviewed all answers to the open-ended questions and classified them as meaningful or not meaningful. Reasoning that participants who did not answer the long-answer in a meaningful way were less likely to answer the short-answers meaningfully, we adopted a conservative approach and eliminated participants with “not meaningful” answers, leaving 123, or 70.3% of the original sample. 

Demographics: Table 1 shows the demographic and descriptive characteristics of the sample for both Study 1 and Study 2. While there was good distribution within demographic variables, the majority of the sample were white, female, and aged 30-44. Twelve percent of the sample lived alone. The size of participants’ hometowns was well distributed with the median size between 30,000 and 60,000 and the mode being cities of greater than 125,000. In Study 2, we collected data on military service (counting both active duty and Veteran status), which found that 8.3% had served (7.5% of females and 10.1% of males). 

Survey and measures: The survey included the three-item UCLA Loneliness Scale to measure loneliness, which is scored on a three-point Likert scale from Hardly Ever to Often. This scale correlates .82 with the full 20-item UCLA loneliness scale [26]. Both have been found to be valid and reliable and are widely used in research on loneliness.

Participants were provided the following definition of acquaintances. “Acquaintances are people you know who are neither friends nor family. They may be people you see because of their role like a mail carrier, a cashier, a waitress or a bus driver. They may be co-workers or neighbors.” They were then asked “In a typical week, about how many times do you have contact with acquaintances, whether in person, by telephone or by video conferencing such as FaceTime or Zoom, etc.? Do not count contacts by text, email or other apps that don't involve talking.” In a separate question, they were asked “In a typical week, about how many times do you have contact with acquaintances, by text, email or other apps that don't involve talking?” Response choices ranged from zero to more than 15. 

Participants were then asked, “About how much of the time do interactions with your acquaintances leave you feeling better?” Ratings were on a five-point scale anchored by "A great deal" and "None at all." In a separate question, they were asked “About how much of the time do interactions with your acquaintances leave you feeling worse?” Ratings were on the same five-point scale. 

Study 2

In Study 2, we repeated the analyses from Study 1 with a new and larger sample and added a measure of social anxiety. Social anxiety is a common clinical concern that, like loneliness, could be a barrier to using casual contacts for an intervention. Social anxiety is a predictor of loneliness [27] and one of its central features, fear of negative evaluation, is also correlated with loneliness [20]. 

Hypotheses

1. As in Study 1, reported interaction with casual contacts will be common across a sample of adults (at least 50% will have one or more weekly) and will be frequently associated with reports of a positive impact (at least 50% will report casual contacts are positive a moderate amount of the time).

2. Participants who report higher levels of loneliness will be more likely to have a negative reaction to casual contacts than their nonlonely peers (replicating Hypothesis 2 from the first study).

3. Participants who report higher levels of social anxiety will be more likely to have a negative reaction to casual contacts than their nonsocially anxious peers.

We also explored how loneliness and social anxiety affect how long both better and worse feelings endure after casual contacts.

Method Study 2

Participants and procedures: We recruited a convenience sample of English-speaking adults living in the US, via a panel from Survey Monkey in December 2022 and August 2023. There were no selection criteria beyond (a) language and location and (b) the sample being generally balanced for age and gender. Participants either received direct compensation or directed funds to a charity of their choice. For Study 2, six additional short-answer questions addressing social anxiety were added to the items in Study 1. As in Study 1, we eliminated all participants whose answers to the open-ended question were not meaningful. From 372 original participants, 270 or 72.6% had meaningful answers. 

Data analysis: Hypothesis 1. Descriptive statistics were used to show both the frequency of casual contacts and how often and to what extent they were associated with positive outcomes. Hypothesis 2.The association of loneliness with negative reaction to casual contacts was assessed by (a) partial correlation and (b) an analysis of covariance (ANCOVA) with loneliness as the independent variable and frequency of feeling worse after casual contacts as the dependent variable. We first ran the ANCOVA with the trichotomized loneliness scale to replicate the analysis in Study 1. We then ran an ANCOVA with the full seven-point loneliness scale. Hypothesis 3. We assessed whether participants with loneliness and social anxiety were more likely to have a negative reaction to casual contacts than their peers without these conditions using ANCOVA with loneliness and social anxiety as the independent variables and frequency of feeling worse after casual contacts as the dependent variable. The relationships of loneliness and social anxiety with how long negative feelings endured after a casual interaction were explored with partial correlations. For all partial correlations and ANCOVAs, five variables were controlled for age, gender, household size, household income, and size of town. 

Table 1 includes demographic data for the sample from Study 2. The sample was primarily white and middle-aged, with more women than men. More than 90% lived with at least one other person.

Measures: To the measures used in Study 1, we added the six-item Social Interaction Anxiety Scale Short Form (SIAS) [28], which has been found to be valid, reliable, and widely used in research. Each item is scored on a five-point Likert scale from "Not at all" to "Extremely." For individuals who were neither working nor in school (that status was obtained in a separate question), we multiplied the sum of the first five items by 1.2 to align with norms of the six-item scale. In addition, we added a question on military service.

In the second wave of data collection for Study 2, participants reported how long (a) better feelings endured and (b) worse feelings endured (on a five-point scale ranging from less than 30 minutes to more than one day) after casual contacts.

## Results

Study 1

Hypothesis 1

Ninety percent of participants reported talking with an acquaintance at least once per week, with a median of four contacts per week. Turning to electronic contact with acquaintances, 91% have contact, with a median of four times per week. Just 3.3% of participants have neither weekly verbal nor electronic contacts with acquaintances. 

Table 2 shows the responses to casual contacts. The majority of respondents reported a positive response, with 83% of participants reporting “feeling better” at least a moderate amount of the time and 47.2% feel better “a great deal” or “a lot” of the time. A smaller portion reported negative reactions to casual contacts, with 35.8% reporting “feeling worse” at least a moderate amount of the time, and 18.7% reporting “feeling worse” a great deal or a lot of the time.

**Table 2:** Valence and intensity of feelings after casual contact: Study 1 and Study 2

Study 1	Better		Worse	
Response	n	%	n	%
A great deal	28	22.8	10	8.1
A lot	30	24.4	13	10.6
A moderate amount	44	35.8	21	17.1
A little	19	15.4	41	33.3
None at all	2	1.6	38	30.9
Study 2				
Response				
A great deal	37	13.7	10	3.7
A lot	71	26.3	24	8.9
A moderate amount	88	32.6	40	14.8
A little	59	21.9	98	36.3
None at all	15	5.6	98	36.3

Hypothesis 2

**Figure 1:**
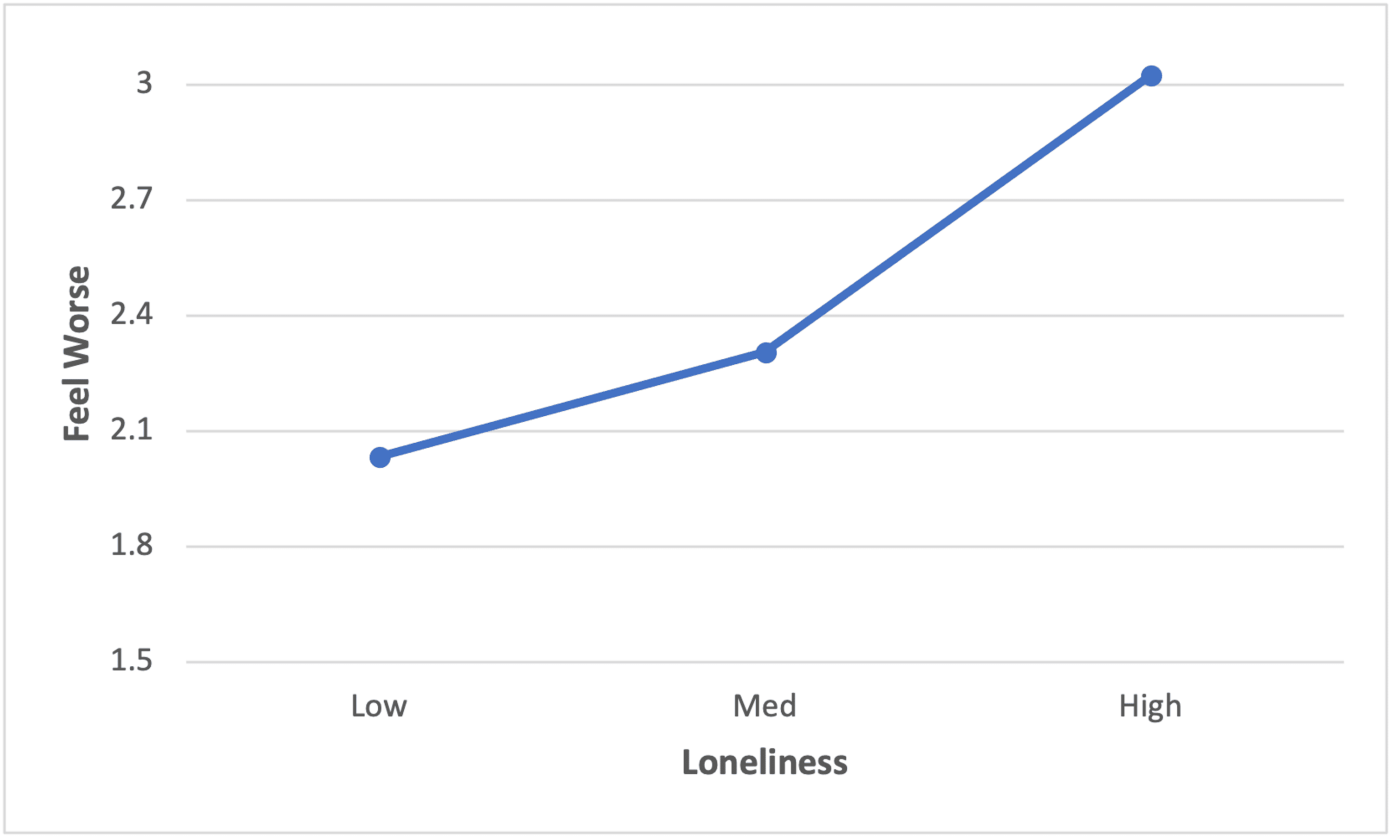
Loneliness by feeling worse after casual contacts: Study 1 (n = 123) Loneliness categories based on the UCLA three-item scale score: low (score 3-4); medium (score 5-7); and high (score 8-9). Loneliness, feel worse partial correlation (r=.25, p < . 01), controlling for age, gender, household size, household income, and size of town

Forty-eight percent of participants reported a moderate level of loneliness (i.e., scores of 5-7 on the UCLA Loneliness Scale) and 15.4% reported a high level, (i.e., scores of 8-9). Figure 1 shows the average frequency that participants feel worse, on a scale of 1 to 5, after casual contacts by the degree of loneliness. Means for feeling worse have been adjusted for age, gender, household income, household size, and town size. The partial correlation of loneliness with "feeling worse" after casual contacts was significant (r = .25, p < .05); the partial correlation with "feeling better" was not significant. 

Using the trichotomized loneliness scale (low, moderate, high) in an ANCOVA that controls for age, gender, household size, household income, and size of town shows that loneliness predicts feeling worse after a casual interaction (F(2) = 4.9, p < .01).

Study 2

Hypothesis 1

Paralleling Study 1, 90.4% of the participants reported talking with an acquaintance at least once per week, with the median number of contacts of four per week. Turning to electronic contact with acquaintances, 86.7% have contact, with a median of four times per week. Just 3.7% of participants have neither weekly verbal nor electronic contacts with acquaintances. The majority of respondents reported a positive response, with 72.6% of participants reporting "feeling better" at least a moderate amount of the time and 40.0% feel better "a great deal" or "a lot of the time." A smaller portion reported negative reactions to casual contacts, with 27.4% reporting "feeling worse" at least a moderate amount of the time, and 12.6% feel worse "a great deal" or "a lot of the time" (Table 2).

Hypothesis 2

**Figure 2:**
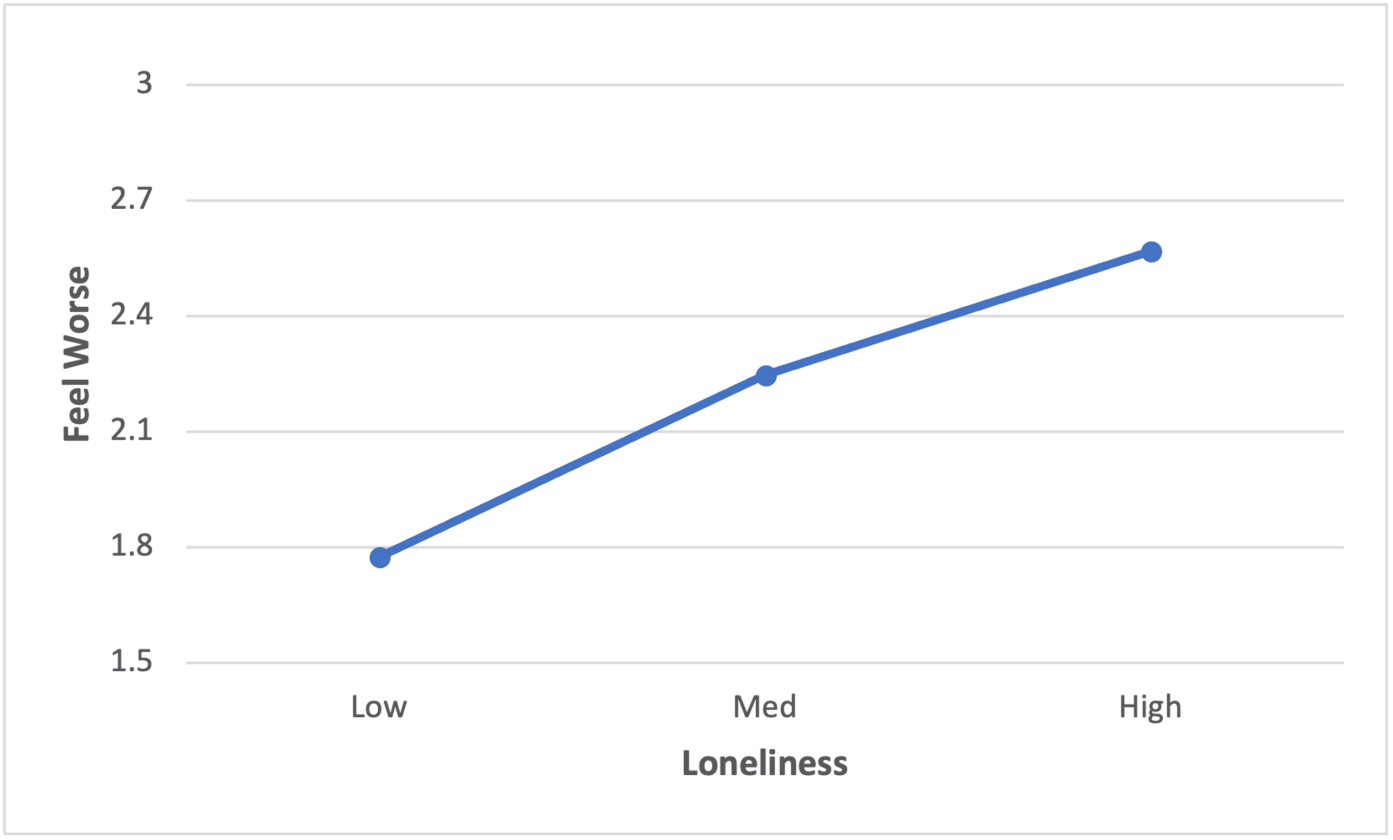
Loneliness by feeling worse after casual contacts: Study 2 (n = 269) Loneliness categories based on the UCLA three-item scale score: low (score 3-4); medium (score 5-7); and high (score 8-9). Loneliness, feel worse partial correlation (r=.30, p < .001), controlling for age, gender, household size, household income, and size of town

Figure 2 shows the average frequency that participants feel worse, on a scale of 1 to 5, after casual contacts by the degree of loneliness. Means for feeling worse have been adjusted for age, gender, income, household size, and town size. As in Study 1, the partial correlation of loneliness with feeling worse after casual contacts was significant (r = .30, p < .001). 

Hypothesis 3

The partial correlations of social anxiety with loneliness (r = .36, p < .001) and "feeling worse" after casual contacts (r = .45, p < .001) were significant. An ANCOVA with social anxiety and loneliness (both trichotomized) as the fixed factors reveals that social anxiety significantly predicts feeling worse after a casual contact (F(2) = 13.4, p <. 001). In addition, the interaction of social anxiety and loneliness is significant (F(4) = 3.1, p < .05). Post-hoc analyses reveal that when social anxiety is high, loneliness does not have an effect on feeling worse after casual contacts. However, when social anxiety is low or moderate, then loneliness significantly increases the risk of feeling worse after casual contacts. Partial correlations and ANCOVAs were all controlled for age, gender, household size, household income, and size of town.

**Figure 3:**
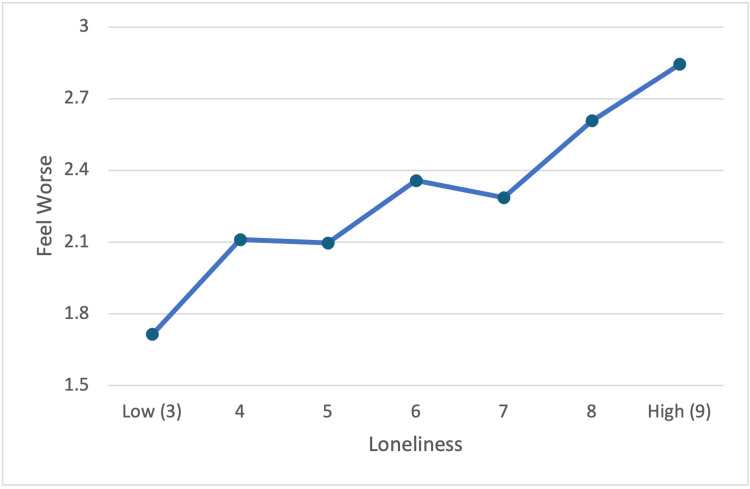
Loneliness by "feeling worse" after casual contacts: total sample-full loneliness scale (n = 392) Loneliness, feeling worse partial correlation (r = .28, p < .001), controlling for age, gender, household size, income, and size of town

Although the UCLA Loneliness Scale scores range from 3 to 9, the number of participants in Study 1 was not large enough to look at the "feel worse" scores separately at each of the seven levels of loneliness. Therefore, we initially divided the loneliness scale into thirds in Study 2 to replicate Study 1’s protocol. Having replicated that "feeling worse" increases with increasing levels of loneliness with the trichotomized scale, we then combined the two samples producing a sample sufficiently large to meaningfully graph the "feel worse" scores at each of the seven levels (i.e., 3-9) of the Loneliness Scale. Figure 3 shows this finer analysis, which again reveals that "feeling worse" scores increase (on a scale ranging from 1 to 5) as Loneliness scores increase (F(6) = 5.9, p < .01). The partial correlation of loneliness with feeling worse was significant (r = .28, p < .001). Again, these analyses controlled for age, gender, household size, household income, and size of town.

We explored whether the length of time feelings endure after casual contacts is affected by the degree of loneliness and/or social anxiety. The partial correlation of both loneliness and SIAS with "feeling worse" for longer periods after casual contacts was significant (r = .16, p < .05; r = .26, p < .001, respectively). The partial correlation of loneliness with the endurance of positive feelings was also significant (r = -.27, p < .001). Age, gender, household size, income, and size of town were controlled in each of these analyses.

## Discussion

The aims of these studies were to determine (a) the emotional responses to casual contacts in a community sample and (b) individual differences in emotional responses between persons with higher levels of loneliness and/or social anxiety compared to counterparts with lower levels. Because this is a study of an unexplored area, we ran two studies to allow for replication of the major hypothesis and controlled for five key variables, age, gender, household size, household income, and size of town. We also explored how long feelings endured after casual contacts among persons who are lonely and/or socially anxious. 

Although methods and measurement instruments differ, our results showing that a large majority of participants report a positive emotional response to casual contacts most of the time are consistent with previous studies. Sandstrom and Dunn [15] found that participants assigned to a social condition who were told to “smile, make eye contact” and converse with the barista at a Starbucks showed significantly more positive affect and less negative affect than those assigned to an efficient condition who were instructed to “avoid unnecessary conversation." Epley and Schroeder [29] found that commuters assigned to a connection condition (connect with someone new, “find out something interesting about” them and “tell them something about you”) reported significantly more positivity than those assigned to a solitude condition (“keep to yourself and enjoy your solitude”). Our results also show a significant minority of participants reported that they had a negative emotional response to casual contacts much of the time. Wesselmann and colleagues [30] found that participants who were unacknowledged by passing strangers felt more disconnected than those who were acknowledged by either eye contact or eye contact accompanied by a smile.

Our findings support the view that individual differences impact the response to casual contacts. Both loneliness and social anxiety increase the likelihood of having a negative response to casual contacts. However, when social anxiety is high, the impact of loneliness on feelings emerging from casual contacts is no longer apparent. Exploratory analyses also showed that for people with high scores on loneliness, positive responses to casual contacts don’t last as long and negative responses last longer. Similarly, increased levels of social anxiety predicted that feeling worse after casual contacts lasts longer. We are unaware of any other work that has studied the impact of loneliness and social anxiety on casual contacts. 

These novel results have important clinical implications. The finding that casual contacts are quite common and most typically result in a generally positive response suggests that contacts with acquaintances, including coworkers, neighbors, and even delivery people, have a positive emotional impact and should be considered a significant part of people’s social network. Efforts to enhance levels of social support should consider this group of contacts along with family and friends. In addition, scales should be broadened or developed to capture this clinically important variable. 

The results related to participants with high scores on the loneliness and/or the social anxiety scales provide a cautionary point: individual differences among people are relevant to their responses to potential interventions using casual contacts. People who are lonely or socially anxious, two groups who would be likely targets for an intervention to build social support, are more likely to find casual contacts to have a negative impact. Intervening to expand their networks to include casual contacts is supported but only if they receive an intervention tailored to their individual vulnerabilities. 

Whether increasing casual contacts that are more rewarding will impact the mood and general well-being of lonely and isolated persons for more than a short time requires further study. Open questions remain. Can casual contacts by themselves improve quality of life? Can they provide a first step toward broader and deeper interactions with others? Future research may also profitably include social isolation and replicate this exploratory work on the short-term duration of feelings after casual contacts.

This research is limited by the use of a convenience sample. While it includes persons from all regions of the US and representation from rural, small town, and urban environments, some groups are underrepresented including minority racial groups identified in the US census. In addition, all participants identified as either male or female, leaving those with other identities unrepresented and males constituted only about 40% of each sample. Finally, four questions used in this survey to measure participants' feelings in response to casual contacts as well as the length of time those feelings endured were used for the first time in this research. Although these items have face validity and results with these items were consistent with theory, they have not been previously validated. 

## Conclusions

This paper confirms previous findings that casual contacts frequently lead to improved feeling states and adds to the literature in this area by showing that individual differences, specifically loneliness and social anxiety, are associated with negative outcomes to casual contacts. Clinicians seeking to use causal contacts to build social support should investigate an individual’s levels of loneliness and social anxiety before making recommendations. Theory suggests that other conditions likely to increase social hypervigilance or social apprehension (e.g., social isolation) should also be assessed prior to recommending a treatment course. When one or more of these conditions are present, preliminary clinical work should include a review of clients’ thoughts about what is required in casual conversations as well as their social skills along with necessary corrective measures. Additional research is necessary to develop a standard protocol to capitalize on these findings.

## Appendices

The survey

Notes: For items 1-10, participants could choose only a single answer, except for question 2 for which they could check all that apply. Questions 11, 12, 13, and 15 each show the instructions to participants. Question 14 allowed a single answer. Gender and age range for each participant were collected by Survey Monkey. There were 10 other questions on the survey that were not part of the research reported in this article and are not shown here or referred to in the Methods section.

1. What is the size of the town/suburb or city in which you live? a. Fewer than 5,000 b. Between 5,000 and 15,000 c. Between 15,001 and 30,000 d. Between 30,001 and 60,000 e. Between 60,001 and 125,000 f.  Greater than 125,000

2. Please check all of the following with which you identify.​​​​​​​ a. White​​​​​​​ b. Black or African American​​​​​​​ c. American Indian or Alaskan Native​​​​​​​ d. Asian​​​​​​​ e. Native Hawaiian or Other Pacific Islander​​​​​​​ f.  Hispanic or Latino

3. Are you currently or have you ever served in the US military?​​​​​​​ a. Yes, active duty/reserves​​​​​​​ b. Yes, Veteran​​​​​​​ c. No, never served​​​​​​​ d. Prefer not to answer

4. How many people, besides yourself, live in your household? a.   No one, I live alone​​​​​​​ b.   1​​​​​​​ c.   2​​​​​​​ d.   3​​​​​​​ e.   4​​​​​​​ f.    5 or more

Acquaintances are people you know who are neither friends nor family. They may be people you see because of their role like a mail carrier, a cashier, a waitress, or a bus driver. They may be coworkers or neighbors.

5. In a typical week, about how many times do you have contact with acquaintances, whether in person, by telephone, or by video conferencing such as FaceTime or Zoom, etc.? Do not count contacts by text, email, or other apps that don't involve talking.​​​​​​​ a. 0​​​​​​​ b. 1​​​​​​​ c. 2​​​​​​​ d. 3​​​​​​​ e. 4​​​​​​​ f.  5​​​​​​​ g. 6-10​​​​​​​ h. 11-15​​​​​​​ i.  More than 15​​​​​​​

6. In a typical week, about how many times do you have contact with acquaintances, by text, email, or other apps that don't involve talking. a. 0 b. 1​​​​​​​ c. 2​​​​​​​ d. 3​​​​​​​ e. 4​​​​​​​ f.  5​​​​​​​ g. 6-10​​​​​​​ h. 11-15​​​​​​​ i.  More than 15​​​​​​​

7. About how much of the time do interactions with your acquaintances leave you feeling better?​​​​​​​ a. A great deal​​​​​​​ b. A lot​​​​​​​ c. A moderate amount​​​​​​​ d. A little​​​​​​​ e. None at all

8. How long does the better feeling last? (This question was only given to participants in Phase 2 of Study 2.)​​​​​​​ a. Less than 30 minutes​​​​​​​ b. 31 minutes to 2 hours​​​​​​​ c. Between 2 and 6 hours​​​​​​​ d. More than 6 hours to the whole day​​​​​​​ e. More than one day

9. About how much of the time do interactions with your acquaintances leave you feeling worse? a. A great deal b. A lot​​​​​​​ c. A moderate amount​​​​​​​ d. A little​​​​​​​ e. None at all

10. How long does the worse feeling last? (This question was only given to participants in Phase 2 of Study 2.) a. Less than 30 minutes​​​​​​​ b. 31 minutes to 2 hours​​​​​​​ c. Between 2 and 6 hours​​​​​​​ d. More than 6 hours to the whole day​​​​​​​ e. More than one day

11. We are most interested in the impact of acquaintances (neighbors, coworkers) including casual contacts (a cashier, a postal worker, a person on the street) that are a part of daily life. Would you please tell us about some that stand out for you, what happened and how it affected you? We are interested in both positive and negative effects. For example, if you regularly chat with your mail carrier, please share how that affects you. We'd like to know whether positive or negative reactions occur more often and how long that feeling might last, minute(s), hours, or even days. (Essay type, long-answer question. Expandable space was provided online for participants’ answers.)

12. These questions are about how you feel about different aspects of your life. For each one, please tell us how often you feel that way. (For each of the following three questions, the user had the choice to answer, “Hardly ever”; “Some of the time”; or “Often”. This is the Three-Item Loneliness Scale [26].)​​​​​​​ a. How often do you feel that you lack companionship?​​​​​​​​​​​​​​ b. How often do you feel left out?​​​​​​​ c. How often do you feel isolated from others?

13. Please indicate the degree to which you feel each statement is characteristic or true of you. (For each of the following six questions, the user had the choice to answer, “Not at all”; “Slightly”; “Moderately”; “Very;” or “Extremely.” These questions were given only to participants in Phase 2. Together with Item 15 (and subject to the note in 150, these five items are the Short Form Social Interaction Anxiety Scale (SIAS-6) [28].)​​​​​​​ a. I have difficulty making eye contact with others.​​​​​​​ b. I tense up if I meet an acquaintance on the street.​​​​​​​ c. I feel tense if I am alone with just one person.​​​​​​​ d. I have difficulty talking with other people.​​​​​​​ e. I find it difficult to disagree with another’s point of view.

14. Which of the following categories best describes your status? (This question was used in this study solely to direct users who answered a, b, e, f, or g to question 15 and to have the others skip that question. See the Methods section for the scoring protocol used.)​​​​​​​ a. Employed and working 40 or more hours per week​​​​​​​ b. Employed and working 1-39 hours per week​​​​​​​ c. Not employed and looking for work​​​​​​​ d. Not employed and NOT looking for work​​​​​​​ e. Full time student​​​​​​​ f.  Part time student​​​​​​​ g. A student AND employed 15 or more hours per week​​​​​​​ h. Retired​​​​​​​ i.  Disabled or not able to work

15. To what degree do you find it difficult mixing comfortably with the people you work or go to school with. (The user had the same choices as in question 13, “Not at all”; “Slightly”; “Moderately”; “Very”; or “Extremely.” These questions were given only to participants in Phase 2. Note that the original item on the SIAS-6 was “I find it difficult mixing comfortably with the people I work with.” Because our previous work had shown that some people had difficulty with this item because they were students or not working, we (a) added “or go to school” to the question itself and (b) checked on participants’ school/work status to present this item to the user only if appropriate.)
